# The Sensory, Chemical and Microbiological Quality of Sous‐Vide Cooked Sea Bass (*Dicentrarchus labrax* Linneaus, 1758) With Liquid Smoke Flavor

**DOI:** 10.1002/fsn3.72184

**Published:** 2026-07-31

**Authors:** Rukiye Köklü, Hülya Turan, Can Okan Altan, Demet Kocatepe, Bayram Köstekli, Bengunur Corapci

**Affiliations:** ^1^ Graduate Education Institute, Department of Fisheries and Processing Technology Sinop University Sinop Türkiye; ^2^ Faculty of Fisheries, Department of Fisheries Fishing and Processing Technology Sinop University Sinop Türkiye

**Keywords:** liquid smoke, sea bass, sensory attributes, shelf life, sous‐vide

## Abstract

This study investigated the sensory, chemical, and microbiological quality characteristics of liquid smoke‐flavored sea bass fillets cooked sous vide at three different temperatures (60°C, 65°C, and 70°C) for 20 min and stored at 2°C ± 1°C for 32 days. The aw value, which is 0.985 in fresh fish, remained unchanged in the SV_60_ and SV_65_ groups after sous vide cooking, while it was found to be 0.981 in the SV_70_ group (*p* < 0.05). Sous vide cooking increased L* (brightness) and b* (yellowness) values in all groups, while a* values decreased (*p* < 0.05). In terms of TBARs values, all groups were found to be of very good quality at the end of storage. At the end of the storage period, the TVB‐N value of all groups remained within acceptable limits. In the SV_60_ and SV_65_ groups, total aerobic mesophilic, psychrophilic bacteria, and yeast‐mold counts exceeded the acceptable limit on day 28, while in the SV_70_ group, mesophilic bacteria and yeast‐mold counts were within acceptable limits, but the psychrophilic bacteria count exceeded the limit. According to the research results, the most preferred product group in terms of sensory characteristics for sous vide cooked sea bass with liquid smoke flavor was SV_65_; the optimum cooking temperature was determined to be 65°C for 20 min, and its shelf life was found to be 24 days when stored at 2°C ± 1°C.

## Graphical abstract

The processing of sea bass fillets, flavored with liquid smoke‐salt‐vegetable oil, using sous‐vide technology is an innovative method that both enhances organoleptic quality and ensures safe preservation. The fillets, which were flavored and vacuum‐sealed, were cooked at different temperatures and then transferred to cold storage following rapid cooling. Analyses carried out during storage have shown that the sous‐vide process slows down microbial growth and preserves the product's freshness. When consumer preference, textural structure, and food safety are assessed together, it has been determined that the ideal cooking condition is 65°C for 20 min and that the product can maintain its quality for 24 days within the cold chain.

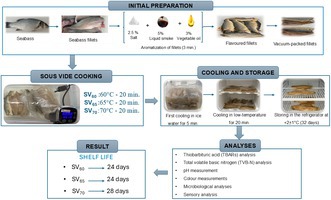

## 1 Introduction

In today's conditions, a significant increase is observed in the production and variety of ready‐to‐eat seafood products. Smoked products, one of these, are highly appreciated due to their aromatic and organoleptic properties. However, in traditional smoking methods, the long processing time and labor intensity, the exposure of the product to high temperatures, and the risk of polycyclic aromatic hydrocarbon (PAH) content are disadvantages for these products. As an alternative, liquid smoking is quite advantageous in terms of cost and time, is easy and practical to apply, and makes liquid‐smoked fish more attractive due to its lower PAH content (Hattula et al. 2001; Alçiçek 2010) and higher ω‐3 and DHA content (Swatawati et al. 2000). Liquid smoking can be combined with many cooking techniques. One of the cooking methods that has come to the forefront in recent years due to its health benefits is sous‐vide cooking. Sous‐vide is defined as “intermediate foods or raw materials cooked under controlled temperature and time conditions in heat‐resistant, vacuum‐sealed bags” (Schellekens 1996). Cooking time can vary from 5 min to 6 h depending on the thickness of the product and the cooking temperature (between 65°C and 95°C). Products that undergo rapid cooling and cold storage after cooking can be heated to 53°C–55°C for consumption (Baldwin 2012). Cook‐chill methods are a technology where foods are generally cooked below 100°C and then the final midpoint temperature of the product is cooled by 0°C–3°C (in ice water) and stored under controlled conditions at 0°C–3°C (Bekhit and Roohinejad 2016). In the sous‐vide method, nutrient loss is minimized because the product is cooked at lower temperatures in vacuum‐sealed packaging (Armstrong 2004), and fat content (Garcia‐Linares et al. 2004), beneficial fatty acids (Ghazala et al. 1996; Stankov et al. 2020; Modzelewska‐Kapituła et al. 2022, 2023), and protein are better preserved (Çetinkaya et al. 2015). This is a significant advantage for consumers who prioritize healthy eating and healthy nutrition. Sous‐vide cooking results in temperature‐specific collagen denaturation, actin solubility, sarcoplasmic protein aggregation, and myofibrillar protein degradation, with thermal myosin denaturation and sarcoplasmic protein aggregation occurring at temperatures above 40°C (Sakuyama et al. 2025). However, cooking losses are greater when sous‐vide is performed at high temperatures (Öz and Seyyar 2016). The shelf life of sous‐vide cooked products in vacuum packaging can vary up to 1 month depending on the fish species, cooking temperature and time, and additional processing (additives, extracts, essential oils, combined processing). Numerous studies have been conducted on the sous‐vide technique; research has focused on the effects of different cooking temperatures and processing times (González‐Fandos et al. 2005; Díaz et al. 2009; Çetinkaya et al. 2015; Öz and Seyyar 2016; Çağlak et al. 2017; Cropotova et al. 2019; Modzelewska‐Kapituła et al. 2022), comparisons with conventional cooking methods (Garcia‐Linares et al. 2004; Wan et al. 2019; Kocatepe et al. 2025), and applications to different fish species (Coşansu et al. 2011; Mol et al. 2012a, 2012b). In addition, the effects of various spices and herbs (Ceylan and Ünal Şengör 2019; Çetinkaya 2020), plant extracts (Alves et al. 2020; Bozova and İzci 2021), and essential oils have been investigated. Sous‐vide processing has also been studied in combination with irradiation (Öztürk et al. 2021), other preservation techniques (Dogruyol and Mol 2017), and high hydrostatic pressure technology (Zhou et al. 2022). Furthermore, the suitability of different fish body parts for sous‐vide processing has been evaluated (Pino‐Hernández et al. 2020). While a small portion of these studies investigated the effects of sous‐vide cooking on nutritional composition, others examined the sensory, chemical, and microbiological quality of the product. Among the studies on sous‐vide, no study combining liquid smoking and sous‐vide technology was found.

Sea bass is one of the most valuable fish due to its high protein and fat content, essential amino acids, valuable minerals, and significant amounts of omega‐3 fatty acids such as EPA and DHA (Kocatepe and Turan 2012). It is a highly preferred fish species by consumers due to its white flesh and flavor. Sea bass, which is farmed in significant quantities in net cages in the sea in Türkiye, is generally exported fresh or frozen to many countries in the world, mainly European countries. For consumers who prioritize healthy eating, minimally processed, ready‐to‐eat seafood is becoming increasingly important. As mentioned above, the unique taste and aroma of smoked products are attractive to consumers. Various studies have also revealed the preservative effects of liquid smoke on the product. Therefore, this study aims to produce a ready‐to‐eat product by integrating liquid smoke, known for its smoky flavor and preservative properties, with the sous‐vide cooking technique, thereby providing consumers with a healthier alternative. As sous‐vide processing is performed under vacuum conditions, it enables uniform cooking at relatively low temperatures and short cooking times. Sea bass possesses a muscle structure that is rapidly cooked owing to its high‐moisture content and low connective tissue content. Therefore, a fixed cooking time of 20 min and three cooking temperatures within a limited temperature range (60°C, 65°C, and 70°C) were employed in this study. The objective of this study was to evaluate the sensory, chemical, and microbiological shelf life of liquid smoke‐flavored sea bass cooked by the sous‐vide method at these temperatures and stored at 2°C ± 1°C.

## 2 Materials and Methods

### 2.1 Material

A total of 38 kg of sea bass (*Dicentrarchus labrax*, Linnaeus, 1758) with an average length of 32.79 ± 0.26 cm were used in the study. The fillets were flavored with iodised table salt, sunflower oil [containing 11% saturated fatty acids (SFA), 35% monounsaturated fatty acids (MUFA) and 53.8% polyunsaturated fatty acids (PUFA)] and liquid smoke named G‐AROME 24 TB FLAVOR with code 152.SMK.6501 [(pH: 2.5–3.5; Acetic acid (%): 7.0–9.0; density (lbs/gal): 9.4–9.6)] were used. For packaging the fillets, 25 × 35 cm, 82‐μm‐thick PE/PA/PE vacuum/film bags and an Abant MG42 brand/model vacuum packaging machine were used, and a Packtech‐IPX7 Sous‐Vide Cooker (PackTech Machine, Egypt) was used for sous‐vide cooking.

### 2.2 Method

#### 2.2.1 Flavoring Step (Salt + Sunflower Oil + Liquid Smoke) and Sous Vide Cooking

The fish were gutted and filleted at a fish processing plant, iced in polystyrene boxes, and transported to the Fish Processing and Quality Control Laboratory within approximately 1 h. All fillets were soaked for 3 min in a mixture of 2.5% salt, 3% sunflower oil and 5% liquid smoke to enhance flavor and aroma in three groups and two replicates. Then, all fillets (2 fillets per package; approximately 200 g) were vacuum‐packed and cooked using the sous‐vide method at 3 different temperatures (60°C, 65°C, 70°C) for 20 min. During cooking, the temperature was monitored with a thermometer every 5 min to ensure the desired temperature was maintained. Midpoint temperature control was performed on all cooked fish. The sous‐vide cooked fillets were first cooled in ice water for 5 min, then in a low‐temperature cooler for 20 min. At the end of the process, all products with an internal temperature of approximately 2.5°C–3°C were stored in a +2°C refrigerator. The groups created in the experiment are presented in the table below (Table 1).

**TABLE 1:** Groups created in the experiment.

Groups	Process
F	Fresh sea bass fillet
LS	The fillets were soaked for 3 min in a mixture of 2.5% salt, 3% sunflower oil and 5% liquid smoke (flavored fillets)
SV_60_	Vacuum‐packed flavored fillets were cooked sous‐vide at 60°C for 20 min and stored at 2°C ± 1°C
SV_65_	Vacuum‐packed flavored fillets were cooked sous‐vide at 65°C for 20 min and stored at 2°C ± 1°C
SV_70_	Vacuum‐packed flavored fillets were cooked sous‐vide at 70°C for 20 min and stored at 2°C ± 1°C

### 2.3 Analyses

Analyses were performed on fresh fish meat, after flavoring, and after sous‐vide cooking. Additionally, all analyses were conducted every 4 days during storage, with 2 replicates and 3 parallels.

#### 2.3.1 pH Measurement

The pH measurement was performed by inserting a pH meter probe into homogenized samples using a device manufactured by Werkstatten 82,362 Weilheim, Germany.

#### 2.3.2 Water Activity (Aw)

The water activity (aw) value of the samples was determined using a Novas LabSwift–aw device. Water activity values were recorded by placing approximately 5 g of homogenized sample into the device's transparent, round sample containers (Horwitz et al. 1980).

#### 2.3.3 Color Measurements

The a* value (redness), b* value (yellowness), and L* value (lightness) measurements of the samples were performed using a Konica Minolta/CR‐A33a device according to the Hunter colourimeter scale (Schubring 2003).

### 2.4 Total Volatile Basic Nitrogen (TVB‐N) Analysis

The Lücke‐Geidel method modified by Antonacopoulas was used to determine the Total Volatile Basic Nitrogen (TVB‐N) content, which plays an important role in determining the freshness of fish (Varlık et al. 1993). 10 g of homogenized sample was placed in a glass flask with 1 g of MgO, a few drops of silicone oil and 100 mL of distilled water. In a separate location, 10 mL of 3% boric acid, 8 drops of Tashiro indicator and 100 mL of distilled water were added to an erlenmeyer flask used for titration. The glass flask containing the sample and the erlenmeyer flask were placed in a position to receive the distillate and subjected to distillation for 15–20 min. The distillate obtained was titrated with 0.1 N HCl until the color changed from green to pink. The TVB‐N amount was calculated according to the following formula:

(1) $$\mathrm{TVB}-N\;\mathrm{mg}/100g=\mathrm{HCl}\;\text{consumed}\times 0.14/\text{sample weight}\;\left(g\right)$$

#### 2.4.1 Thiobarbituric Acid (TBARs) Analysis

The TBARs analysis was performed according to Erkan et al. (2011). 10 g of sample was homogenized with 100 μL butylated hydroxytoluene (BHT) and 90 mL trichloroacetic acid (TCA) using an ultra‐torx for 3 min. The homogenates were filtered through Whatman No. 1 filter paper and 5 mL was transferred to test tubes. Five milliliters of thiobarbituric acid reagent prepared with 10% glacial acetic acid was added and the mixture was incubated in a water bath at 75°C–79°C for 30 min. After the tubes cooled, they were read at 532 nm against a blank in a spectrophotometer. Preparation of Standards: 50 μL of [(MA) Malondialdehyde bis (diethylacetal)] was diluted with 50 mL of 0.1 N HCl. The dilution was heated at 90°C for 10 min. As a result, 2.4 mL of the resulting hydrolysed acetal was added to a 100 mL flask and made up to the mark with distilled water to prepare the stock standard. From the stock standard, 1, 3, 5, and 7 mL were taken into 50 mL volumetric flasks, and each was made up to 50 mL with distilled water. 5 mL was taken from each standard, 5 mL of TBA reagent was added, and the tubes were incubated in a hot water bath at 75°C–79°C for 30 min. After the tubes cooled, the values read at 532 nm against the blank in the spectrophotometer were used to calculate the regression equation of the standards, and the TBARS (μg malonaldehyde (MA)/mL) concentration was determined. The TBARS values in the samples were calculated according to the following formula:

(2) $$\text{TBARS}\;\left(\mathrm{\mu g}\;\mathrm{MA}/g\right)=\mathrm{MA}\;\left(\mathrm{\mu g}\;\mathrm{MA}/\mathrm{mL}\right)\times 90\;\mathrm{mL}/\text{sample weight}\;\left(g\right)$$

(3) $$\mathrm{MA}\;\left(\mathrm{\mu g}\;\mathrm{MA}/\mathrm{mL}\right):\text{absorbance of the sample}-a/b\;\left(90:\text{dilution factor}\right)$$

#### 2.4.2 Microbiological Analyses

10 g of homogenized fish meat was mixed with 90 mL of 0.85% sterile physiological saline. Microbiological cultures were performed using the pour plate method with the resulting dilutions. Plate Count Agar (PCA) was used for the total mesophilic and psychrophilic aerobic bacteria analysis; the petri dishes were incubated at 37°C for 1 day for mesophilic aerobic bacteria and at 7°C for 10 days for psychrophilic bacteria. Reinforced Clostridial Agar (RCA) was used for total anaerobic bacteria analysis, and the petri dishes were incubated at 37°C for 1 day. Potato Dextrose Agar (PDA) was used for total yeast and mold counting, and the petri dishes were incubated at 25°C–28°C for 4–5 days. Violet Red Bile Agar (VRBA) was used for total coliform bacteria count, and the petri dishes were incubated at 37°C for 1 day (Halkman 2005).

### 2.5 Sensory Analysis

A sensory evaluation form (Table 2), modified from Pons‐Sánchez‐Cascado et al. (2006) and Turan and Kocatepe (2013), was used for sensory analysis. A hedonic scale of 0–10 was used in the sensory analysis form to describe the characteristics of the cooked fish. A score of 10–9 was rated as “extremely liked”; 8–7 as “liked”; 7–6 as “neither liked nor disliked”; 5 as “slightly disliked”; and 4 and below as “extremely disliked”. The Sensory Evaluation Committee established by the Institute of Food Technologists in the USA states in its sensory analysis guide that 3–10 trained, 8–25 semi‐trained, and > 30 untrained panelists can be used in panels (Altuğ Onoğur and Elmacı 2011). Consequently, five trained panelists were assigned to the sensory panel, and sensory analysis was conducted with two replicates for each group (*n* = 10). Panelists with prior sensory evaluation experience (> 10 trials), knowledge of fish freshness, regular fish consumption (≥ 2 times/week), and no fish allergies were selected. Gender balance was ensured. No additional training was conducted due to prior experience; however, panelists were briefed on the study objectives. Sensory evaluations were performed individually under room temperature conditions without interaction among panelists. Sensory panel consent forms were obtained from each panelist participating in the sensory analysis.

**TABLE 2:** Sensory evaluation criteria for liquid smoke‐flavored, sous‐vide cooked fish.

Score/Criterion	Odor	Taste and juiciness	Texture	Aroma	Appearance
10	Characteristic smoked fish aroma	Characteristic smoked fish flavor, very juicy	Smooth and firm	Liquid smoke aroma is highly noticeable	The surface of the fish meat is bright with a smoky color, the underlying texture is white and bright
9	Characteristic smoked fish aroma, mild	Characteristic smoked fish flavor, juicy	Partially smooth and firm in texture	The liquid smoke aroma is moderately noticeable	The surface of the fish meat is bright, smoke‐colored, and the underlying tissue is white and shiny.
8	Characteristic smoked fish aroma, mild	Characteristic smoked fish flavor, juicy	Very slightly firm	The liquid smoke aroma is faintly detectable	The surface of the fish meat is slightly shiny with a smoky color, the underlying texture is white and slightly shiny.
7	Characteristic smoked fish aroma has diminished, partially pleasant	Characteristic smoked fish flavor is diminished, with a slightly unpleasant taste, partially moist	Very slightly firm	Liquid smoke aroma is not detectable.	The surface color of the fish flesh is brown and dull, the underlying texture is white and slightly shiny
6	Slightly sour, fruity odor	Partially metallic, sour taste, dry	Fibrous texture	Liquid smoke aroma is spoiled	Fish meat surface color is dark brown and dull, underlying texture is light yellow and dull
5	Off fishy odor	Intense metallic and sour taste, dry	Soft texture	Bitter smoke	Fish flesh surface color dark brown, underlying tissue yellow
4	A strong, unpleasant fishy odor	Bitter taste	Complete deterioration in texture	—	The surface color of the fish flesh is dark brown and dull, with the underlying tissue being yellow and dull
3	Lactic acid odor, strong ammonia odor	—	—	—	—
2	Ammonia, sulfite odor	—	—	—	—

### 2.6 Statistical Analysis

The data obtained at the end of the study were evaluated using the Minitab (Ver. 21.1) software package with One‐Way ANOVA at a 95% confidence interval. The Tukey test was used to determine the significance level of differences within and between groups. Figures and tables were prepared using MS Office 2020 software and Origin Pro (Vers. 2025) programme.

## 3 Results and Discussion

### 3.1 Water Activity (a_w_)

**FIGURE 1:**
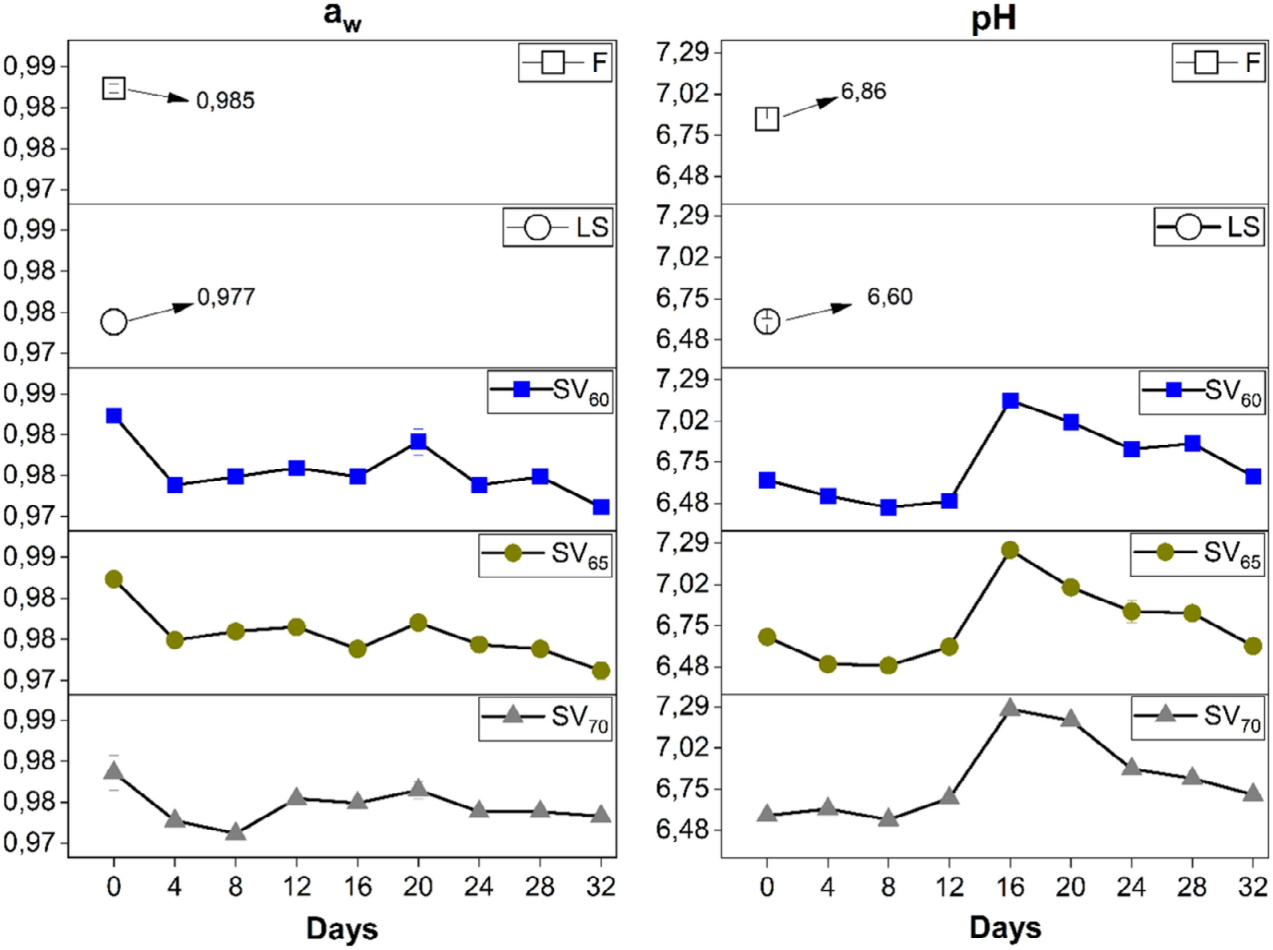
Water activity (a_w_) and pH values of fresh (F), flavored (LS), and sous‐vide cooked fillets (SV_60_, SV_65_, SV_70_).

Water activity is expressed as the ratio of the vapor pressure of water in food to the vapor pressure of pure water at the same temperature, or as the relative humidity of the air in equilibrium with the food. Water activity ranges from 0 to 1. Any factor that causes a change in the vapor pressure of water in food also causes a change in water activity. As the amount of salt, sugar, protein, etc., added to the environment increases, the water activity decreases (Demirci 2012). Water activity (a_w_), which plays an active role in the development of microorganisms and biochemical reactions, is close to 1 in fresh products and decreases to varying degrees with various processing methods such as salting, smoking, freezing, and marinating. In the study, the a_w_ value of 0.985 in fresh fish. The a_w_ value decreased to 0.977 after combined flavoring step (salt + sunflower oil + liquid smoke) (*p* < 0.05) (Figure 1). In this study, the use of 2.5% salt was purely for flavor and was found to be ineffective in reducing water activity. The a_w_ value (0.985) of the groups cooked sous‐vide at 60°C and 65°C on day 0 was like the a_w_ value of fresh fish meat (F) (0.985) (*p* > 0.05). The a_w_ value (0.981) of the group cooked at 70°C was found to be statistically similar (*p* > 0.05) to the aw values of both the flavored group (LS; 0.977) and the other two groups (SV_60_, SV_65_; 0.985). The reason there was no significant difference in water activity measurements between the groups can be explained by the fact that the salt concentration used was not high enough to significantly alter water activity and the sous vide cooking temperatures applied were similar. Minor changes in the a_w_ value were detected in each group during storage, but the differences between groups were found to be insignificant (*p* > 0.05). Since the water content in the fish meat was preserved by sous‐vide cooking in the package, water activity values were measured between 0.985 and 0.974. During the storage, the fact that sous‐vide cooking did not cause a significant change in water activity indicates that the water in the fish meat did not separate from the tissue which largely shows that the textural structure of the fish meat was positively affected. As in this study, findings that sous‐vide cooking does not cause a significant change in water activity have also been reported by González‐Fandos et al. (2005) and Díaz et al. (2011).

### 3.2 pH

The initial pH of fresh fish is 6.86, while the pH of the liquid smoke used in flavoring is 2.65. Due to the acidic properties of the liquid smoke and the effect of salt, the pH of the fish decreased to 6.57 after the flavoring process (Figure 1). Racioppo et al. (2023) also reported that the pH of fresh sea bass (6.10) decreased (5.70) after the liquid smoking process. The pH values of the samples increased because of heat during sous‐vide cooking. Utama et al. (2017) also reported that the pH of meat increased because of thermal denaturation of muscle proteins and lipid oxidation. As reported by Çetinkaya (2020), the pH values of the samples increased because of heat during sous‐vide cooking. Like this study, it has been reported that the pH value measured in fresh perch fillets (6.28) increased after sous‐vide processing at 60°C, 70°C, and 80°C (6.64; 6.43; 6.38) (Şişmanlar Altıkaya 2016). In largemouth bass ( *Micropterus salmoides* ), an increase in pH value (from 6.69 to 6.94) was also reported after a 20‐min sous‐vide application at 85°C (Wan et al. 2019). During sous‐vide cooking and subsequent storage under cold conditions, pH values in all groups ranged between 6.45 and 6.69 until day 12 but were found to be 7 or above on days 16 and 20 (Figure 1). The increase in pH observed on day 16 can be attributed to the increased microbiological load in the product. As seen in Figure 4, microbial growth, which was slow until day 12, is noticeable on day 16, particularly with an increase in total coliform and total anaerobic bacteria counts. However, the microbial load remained stable after day 16 and stayed within acceptable limits. The pH values measured after the 24th day remained between 6.62 and 6.88 in all groups (*p* > 0.05). Consistent with our findings, an increase in pH value has also been reported during cold storage (Garcia‐Linares et al. 2004; Mol et al. 2012b; Şişmanlar Altıkaya 2016; Ceylan and Ünal Şengör 2019) and that this increase may be due to volatile bases produced by microorganisms and enzymes (Ceylan et al. 2018).

### 3.3 Color

**FIGURE 2:**
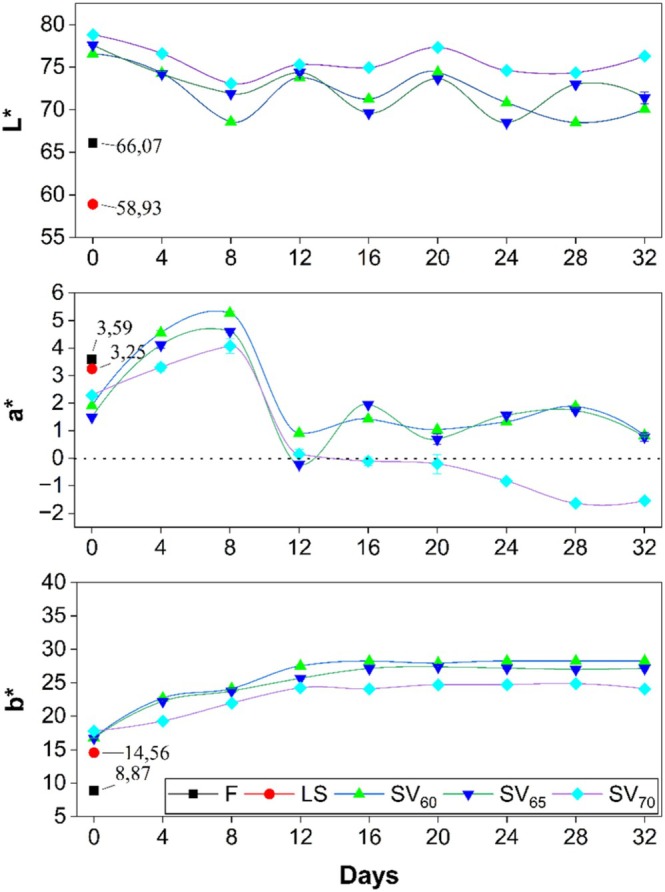
L*, a*, b* values in fresh (F), flavored (LS) and sous‐vide cooked fillets (SV_60_, SV_65_, SV_70_).

The color (L*, a*, b*) values of fresh fish fillets, flavored fillets, and sous‐vide‐cooked fillets are shown in Figure 2.

According to the CIE color system, the L* value used in defining color values indicates surface brightness, while the a* value, which is related to the amount of oxymyoglobin in the meat, is associated with red color, and this value decreases as the meat pigments undergo denaturation during heat treatment. The b* value, which is the yellowness value, is associated with the formation of metmyoglobin and the development of a brownish color because of metmyoglobin denaturation by heat. It is stated that while the L* and b* values increase in meats cooked by the sous‐vide method, the a* value decreases. This situation is explained by the conversion of the color pigment in the meat into the myoglobin form due to vacuum application, and the fact that this form is more resistant to heat than oxymyoglobin (Derin and Serdaroğlu 2020). The L value, determined as 66.07 in fresh sea bass fillet (F), decreased to 58.93 after combined flavoring step (salt + sunflower oil + liquid smoke) (LS), due to the dark color of the liquid smoke. Subsequently, the L values of all groups increased with the sous‐vide cooking process. Thanks to the homogeneous heat distribution in the sous‐vide cooking method, the entire meat becomes evenly light in color and achieves a higher gloss level. A decrease in the L* values of all groups was observed until the 8th day of storage. During this process, chemical changes in protein and fat structure and an increase in bacterial load may have caused discoloration and dulling on the fish surface. As seen in the graphs, there was a significant increase in TBARs values, especially in psychrophilic bacteria counts, during the first 8 days of storage. Slight fluctuations in the L* values detected in the following days were observed. The L* value of the SV_70_ group was found to be higher than that of the other groups throughout the storage period (*p* < 0.05) (Figure 2). The redness value (+a*) of fresh fish fillet, which was +3.59, decreased due to the combined flavoring process and sous‐vide cooking at all three temperatures (*p* < 0.05). This is because, during cooking in a vacuum‐packed container, the myoglobin that gives the meat its red color denatures due to the heat. The +a* value, which increased in all three groups on the 4th and 8th days of storage, decreased again on the 12th day (*p* < 0.05). This situation can be explained by the biochemical changes occurring in the meat structure during this process and a decrease in L* values was also observed during the first 8 days of storage. In the SV_70_ group, which was sous‐vide cooked at 70°C the −a* value, indicating green tones in the fillet color, increased throughout storage, reaching‐1.54 from the 16th day onwards. A negative a* value indicates that the color has shifted toward the green axis rather than the red axis. However, an a* value of −1.54 for fish fillet does not mean that the fillet is visibly “green.” It merely indicates a slight shift in the color coordinate toward the green direction. Therefore, a shift of the a* value from positive to negative, or becoming more negative, indicates a decrease in redness. This situation is often associated with oxidation, pigment changes, or quality changes occurring during storage. The change in the +a* values of the fillets in the SV_60_ and SV_65_ groups cooked sous‐vide at 60°C and 65°C was generally similar, except for the −a* value of the SV_65_ group on the 12th day (Figure 2). The yellowness (+b*) value of fresh fish fillet increased significantly under the effects of combined flavoring step and sous‐vide cooking (*p* < 0.05). The change in the +b* values of groups SV_60_ and SV_65_ was insignificant from the 16th day onwards (*p* > 0.05). Lower +*b* values were observed in group SV_70_, which was sous‐vide cooked at a higher temperature, compared to the other two groups during storage (*p* < 0.05) (Figure 2).

The effect of sous‐vide cooking on L*, a* and b* values was similar in the groups cooked at 60°C and 65°C, but significantly different in the group cooked at 70°C (Figure 2). However, the decrease in a* values and the increase in L* and b* values observed at all temperatures were consistent with various sous‐vide research results (Seyyar 2015; Wan et al. 2019; Modzelewska‐Kapituła et al. 2023). Dong et al. (2017) also reported a decrease in redness (a*) and an increase in yellowness (b*) with sous‐vide cooking, but no significant change in brightness (L*) values. The color development of seafood cooked using sous‐vide technology can be influenced by different cooking temperatures and times. Furthermore, the use of colored sauces and their combination with different antimicrobial components also significantly affects color change (Cui et al. 2022). As Modzelewska‐Kapituła et al. (2023) also stated, the increase in brightness resulting from the cooking of muscle tissue is due to protein denaturation and decreased solubility, while color changes are due to myoglobin denaturation and oxidation. In this study, the detection of higher L* values in the SV_70_ group, which had a higher temperature than the other two groups, indicates that protein denaturation was greater in this group. The changes in redness between raw and cooked fillets after cooking can be explained by the thermal denaturation of myoglobin and hemoglobin in the muscle tissue and the partial removal of pigment‐containing cell fluids during loss of cooking (Modzelewska‐Kapituła et al. 2023).

### 3.4 Total Volatile Basic Nitrogen (TVB‐N)

**FIGURE 3:**
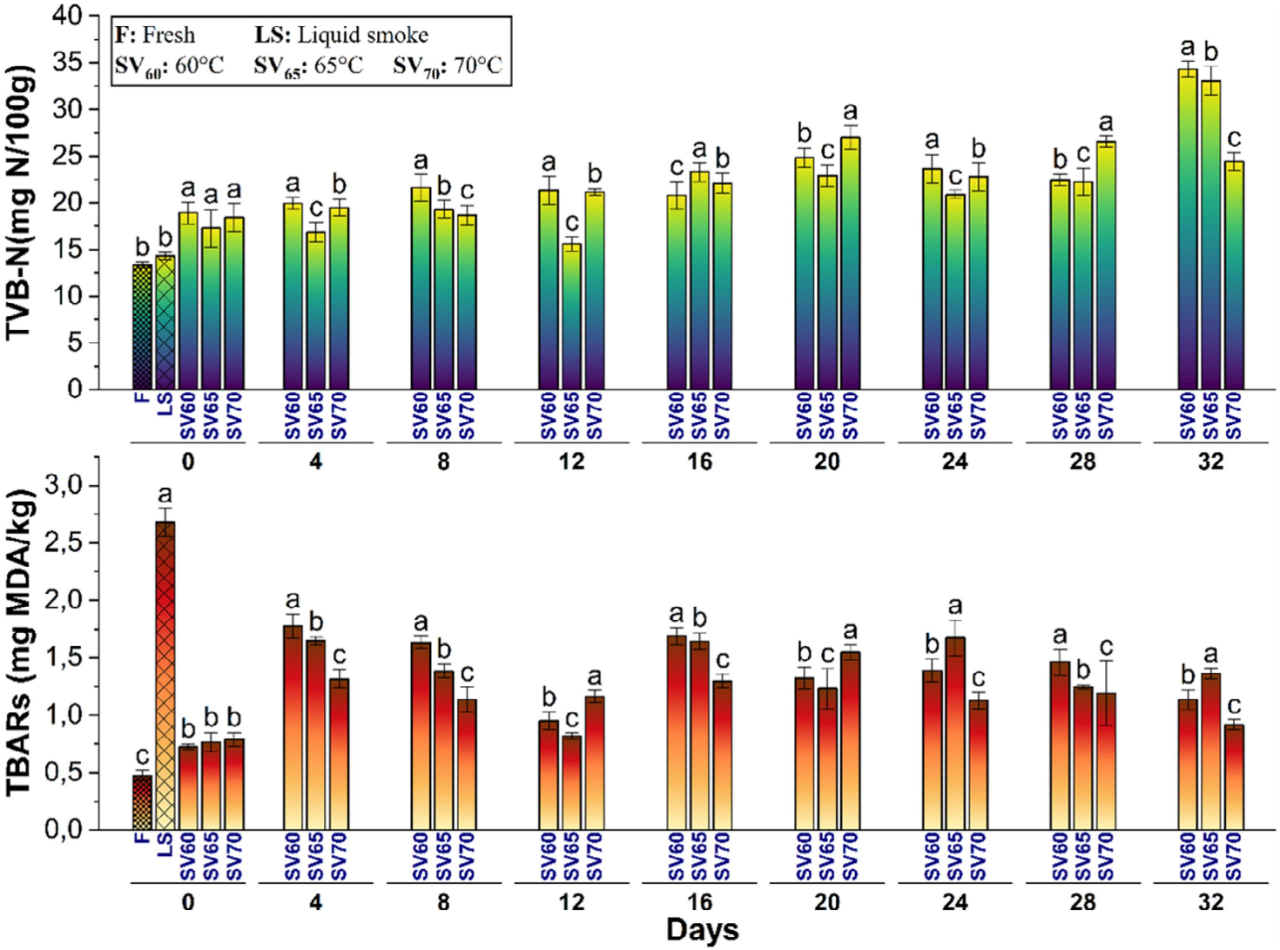
TVB‐N and TBARs values during storage time in fresh (F), flavored (LS), and sous‐vide cooked fillets (SV_60_, SV_65_, SV_70_). (a–c) (→): The difference between groups carrying different letters on each storage day is significant (*p* < 0.05).

Seafood is classified as “very good” if the TVB‐N value, used as a quality criterion, is 25 mg/100 g or below, “good” if between 25 and 30 mg/100 g, “marketable” if between 30 and 35 mg/100 g, and “spoiled” if above 35 mg/100 g (Kietzmann et al. 1969). The TVB‐N content of the fresh sea bass used in the study (13.33 mg/100 g) increased insignificantly (14.32 mg/100 g) after combined flavoring step (salt + sunflower oil + liquid smoke) (*p* > 0.05). Subsequently, there was a significant increase (*p* < 0.05) in the TVB‐N value in the fillets cooked sous‐vide at different temperatures, but the three groups were similar in terms of TVB‐N value (*p* > 0.05) (Figure 3). During the first 28 days of storage, the SV_60_ and SV_65_ groups cooked sous‐vide at 60°C and 65°C were of very good quality, while the SV_70_ group cooked at 70°C was of good quality (Figure 3). The TVB‐N content of all groups did not exceed 35 mg/100 g during storage. Similar to this study, it has been reported that TVB‐N values increased in products cooked sous‐vide and stored cold (Mol et al. 2012b; Çağlak et al. 2017; Russo et al. 2023), while they were relatively lower in those cooked at high temperatures (Şişmanlar Altıkaya 2016; Çağlak et al. 2017; Erümit and Yıldız 2022). It has been reported that the increase in TVB‐N is due to volatile basic compounds formed as a result of the metabolic activity of microorganisms and that the increase is consistent with the growth of microorganisms (Qiu et al. 2014). In this study, a relationship was also observed between TVB‐N change during storage and the growth of microorganisms, with a significant increase in these values from the 16th day onwards. Very similar to this research results, Furiski et al. (2025) found that the sous‐vide process reduced microbial growth in mackerel ( *Scomber colias* ) (mesophilic and psychrophilic abundances remained below 7 LogCFU/g from day 7 to day 21 compared to raw and marinated fillets), and that it reduced the levels of spoilage indicators such as TVB‐N, which remained within acceptable limits of 25–35 mg N/100 g until the 28th day of storage.

### 3.5 Thiobarbituric Acid (TBARs)

Thiobarbituric acid value (TBARs) is an index of secondary lipid oxidation that measures the amount of malondialdehyde (MDA), an oxidation product formed during the oxidation of polyunsaturated fatty acids (Fernández et al. 1997). The TBA value should be less than 3 in a very good product, 3–5 in a good product, and no more than 8 in a marketable product (Varlık et al. 1993). The TBARs value of the fresh sea bass used in this study is 0.48 mg MDA/kg (Figure 3). The TBARs value of the liquid smoke used for flavoring is 27.71 mg MDA/kg. After flavoring (salt + sunflower oil + liquid smoke), the TBARs value of the fish meat increased to 2.68 because of the liquid smoke (*p* < 0.05). With the effect of sous‐vide cooking, the TBARs value decreased, and it was determined that the effect of temperature increase on the TBARs value was insignificant (*p* > 0.05) (Figure 3).

Like this study, Pongsetkul et al. (2022) also found similar TBARs content in groups cooked using the sous‐vide method at different temperatures (*p* > 0.05). As in other studies on this subject (Díaz et al. 2011; Çetinkaya et al. 2017; Bozova and İzci 2021), in this study observed an increase in the TBARs values of the groups during storage (*p* < 0.05). Significant differences (*p* < 0.05) were found in TBARs values between the groups, with the highest TBARs value (1.78 mg MDA/kg) detected in 60°C group (SV_60_). Like the TVB‐N results, lower TBARs values were obtained at the end of the storage period in the 70°C group (SV_70_), which was processed at a higher temperature (Figure 3). This can be explained by the malondialdehyde formed at high temperatures participating in further oxidation reactions or interacting with proteins to transform into compounds that cannot be measured by the TBARs method. This result is like that found by Seyyar (2015) in rainbow trout cooked sous‐vide at medium cooking levels at 65°C, 75°C, and 85°C for 90, 75, and 60 min, respectively. At the well‐done level (150, 135, and 120 min at 65°C, 75°C, and 85°C, respectively), increased cooking times at high temperatures resulted in higher TBA values (Seyyar 2015). This indicates that prolonged cooking at high temperatures has an enhancing effect on fat oxidation. In this study, lower TBA values were obtained with a cooking temperature of up to 70°C and a cooking time of 20 min (Figure 3). The fact that the highest TBARs value detected in this study was below 3 indicates that the product is of very good quality. One of the most important reasons for this is that the fillet was cooked and stored in vacuum packaging. This protected the product from atmospheric air, one of the most important factors causing oxidation. Similarly, it has been reported that TBA values remained within the limit values during 63 days of storage in pikeperch ( *Sander lucioperca* ) cooked sous‐vide at 60°C, 70°C, and 80°C (Çağlak et al. 2017). Wan et al. (2019) also reported that vacuum steaming or sous‐vide cooking methods showed lower lipid oxidation in large‐mouthed bass compared to traditional cooking methods (boiling, steaming). Therefore, due to the lower levels of lipid oxidation occurring in sous‐vide cooked products, the product is of better quality.

### 3.6 Total Mesophilic Aerobic Bacteria

**FIGURE 4:**
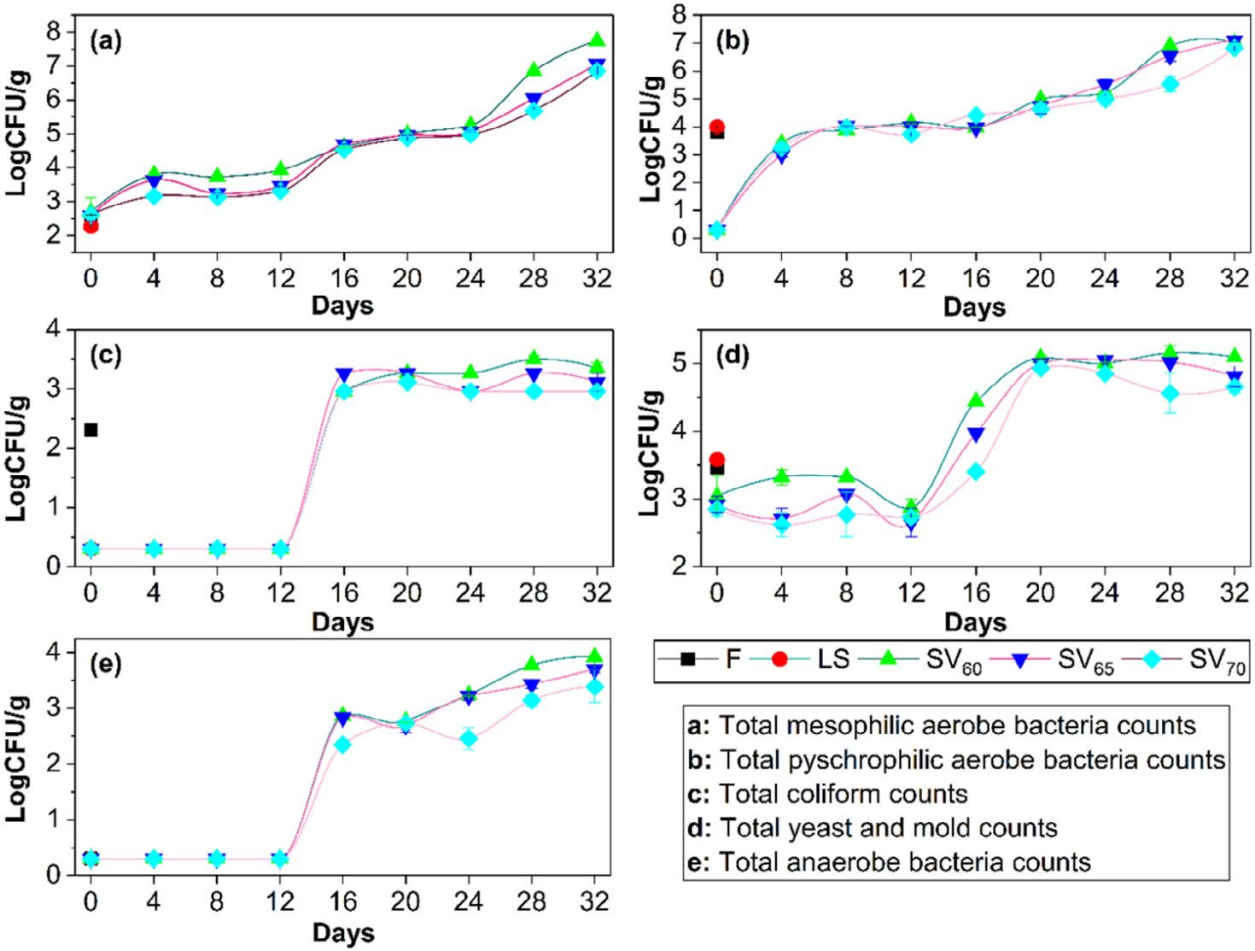
Microorganism counts during storage time in fresh (F), flavored (LS), and sous‐vide cooked fillets (SV_60_, SV_65_, SV_70_).

The total mesophilic aerobic bacteria load of fresh fish (F) was determined to be 2.43 LogCFU/g (Figure 4). Following flavoring step (LS), the bacterial count was 2.27 LogCFU/g, and after sous‐vide processing, it was determined to be 2.68, 2.59, and 2.61 LogCFU/g in groups SV_60_, SV_65_, and SV_70_, respectively (*p* > 0.05). During storage, an increase in the total mesophilic aerobic load was observed over time in all groups (*p* < 0.05). During the 16–20‐day storage period, the total mesophilic aerobic bacteria count in the groups ranged between 4 and 5 LogCFU/g (Figure 4). An increase in TVB‐N value was also observed on the 16th day of storage. This indicates that endogenous enzymes began to play a more active role and spoilage bacteria began to multiply. The limit value for total mesophilic bacterial load is recommended as 7 LogCFU/g (ICMSF 1986). On the 32nd day of cold storage, groups SV_60_ and SV_65_ (7.74 and 7.05 LogCFU/g, respectively) were found to exceed the limit value. In sous‐vide cooking, it has been reported that the number of mesophilic aerobic bacteria, which decreases with the effect of temperature (González‐Fandos et al. 2005; Ramos et al. 2016; Çağlak et al. 2017), increases with storage time and exceeds 6 LogCFU/g after 45 days (González‐Fandos et al. 2005). In farm‐raised pike (*Sander lucioperca*) fillets cooked sous‐vide (SV) at 65°C for 45 min (SV65), 75°C for 20 min (SV75), and 90°C for 10 min (SV90), total viable bacterial counts, *Enterobacteriaceae, Enterococcus* sp., and *Staphylococcus* sp. were below detectable levels in SV75 and SV90, and showed better sensory quality compared to SV65 (Modzelewska‐Kapituła et al. 2022). Considering the results of research on this subject, we can say that microbial growth in sous vide products depends on the nutritional composition of the fish species, the chosen sous vide cooking temperature, and the cooking time.

### 3.7 Total Psychrophilic Aerobic Bacteria

Psychrophilic bacteria affect the microbial load of cold‐stored fish because they grow at low temperatures. According to the FDA (2013), the limit value for the total psychrophilic aerobic bacteria count is reported as 6 LogCFU/g. The total psychrophilic aerobic bacteria load was determined to be 3.83 LogCFU/g in fresh fish (F) and 3.99 LogCFU/g in flavored fish (LS). With the application of heat treatment, this value decreased to 0.30 LogCFU/g in all groups (SV_60_, SV_65_, SV_70_) (Figure 4). On the 28th day of storage, groups SV_60_ and SV_65_ exceeded the limit value, while group SV_70_ exceeded the limit value on the 32nd day. Similar to this study, it has been reported that the total psychrophilic aerobic bacterial load in perch and trout decreased with sous‐vide cooking and increased during storage, and that the total psychrophilic aerobic bacterial count was higher in those cooked at low temperatures (Şişmanlar Altıkaya 2016; Çağlak et al. 2017; Erümit and Yıldız 2022). For Mediterranean mussels (*Mytilus galloprovincialis*) cooked sous‐vide at different temperatures and times (72°C for 450 s, 80°C for 270 s, 90°C for 90 s, 100°C for 40 s), it has been reported that cooking temperatures of 80°C or higher for Mediterranean mussels (*Mytilus galloprovincialis*) significantly improve microbiological quality and that bacterial counts remain within acceptable limits for human consumption even after 21 days of refrigerated storage (Russo et al. 2023).

### 3.8 Total Anaerobic Bacteria

In raw fish meat, the total anaerobic bacteria count in flavored (salt + sunflower oil + liquid smoke) fillets and sous‐vide cooked fillets during the first 12 days of storage was determined to be ≤ 0.30 LogCFU/g. The increase in total anaerobic bacteria count from the 16th day onwards was similar in groups SV_60_ and SV_65_. At the end of storage, the highest anaerobic bacteria count (3.92 LogCFU/g) was determined in group SV_60_, but the differences between the groups were found to be insignificant (*p* > 0.05) (Figure 4). Consistent with our findings, it has been reported that the number of anaerobic bacteria in fish cooked sous‐vide and stored cold generally begins to increase after 2 weeks of storage (Garcia‐Linares et al. 2004; Coşansu et al. 2011; Mol et al. 2012b).

### 3.9 Total Coliform Bacteria

Coliform bacteria should not be present in aquatic products or should be present in very low numbers (Varlık et al. 1993). The total coliform bacterial load in fresh fish meat (F) was determined to be 2.30 LogCFU/g (Figure 4). This situation may be due to contamination during the filleting of the fish and substances added to the fillet. The coliform bacterial load was reduced to ≤ 0.30 LogCFU/g through the flavoring process using a mixture of liquid smoke, salt and sunflower oil. Subsequently, in fillets cooked sous‐vide and stored under cold conditions, the total coliform count was determined to be ≤ 0.30 LogCFU/g up to the 16th day, and no difference was found between the groups (*p* > 0.05) (Figure 4). The change in the increasing coliform bacteria count after the 12th day of storage was similar until the 32nd day. On the 32nd day, the minimum load was detected in group SV_70_, cooked at the highest temperature, and the maximum load was detected in group SV_60_, cooked at the lowest temperature (Figure 4). Like this study, it has been reported that sous‐vide cooking reduces the coliform bacterial load in tilapia fillets (Alves et al. 2020) and that coliform bacteria become inactive in sous‐vide crayfish (Özturan 2022). It has also been reported that coliform bacteria did not develop during storage in sous‐vide cooked mackerel (Mol et al. 2012a).

### 3.10 Total Yeast and Mold

The acceptable yeast and mold count for cooked and cold‐stored seafood has been reported as < 5 LogCFU/g (FDA 2013). The total yeast and mold count of fresh fish meat (F), which was 3.46 LogCFU/g, increased insignificantly with combined flavoring step (LS) (*p* > 0.05). After sous‐vide cooking, the decreasing yeast and mold count (*p* < 0.05) did not exceed 3 LogCFU/g in all groups (SV_60_, SV_65_, SV_70_) during cold storage until the 12th day (Figure 4).

The total number of yeast and mold spores increasing after the 12th day of storage was found to be similar in all groups except for the 16th and 24th days (*p* > 0.05). The number of yeast and mold spores detected between the 20th and 32nd days of storage was also found to be statistically similar in all groups (*p* > 0.05). In groups SV_60_ and SV_65_, which were sous‐vide cooked at a lower temperature, the threshold value was reached on the 20th day. In group SV_70_, which was sous‐vide cooked at a higher temperature, there was less yeast mold development compared to the other groups (Figure 4). As in this study, the lowest yeast and mold count after 18 days of storage in rainbow trout cooked sous‐vide at 55°C, 65°C, and 70°C was observed in the group cooked sous‐vide at 70°C (Erümit and Yıldız 2022).

**FIGURE 5:**
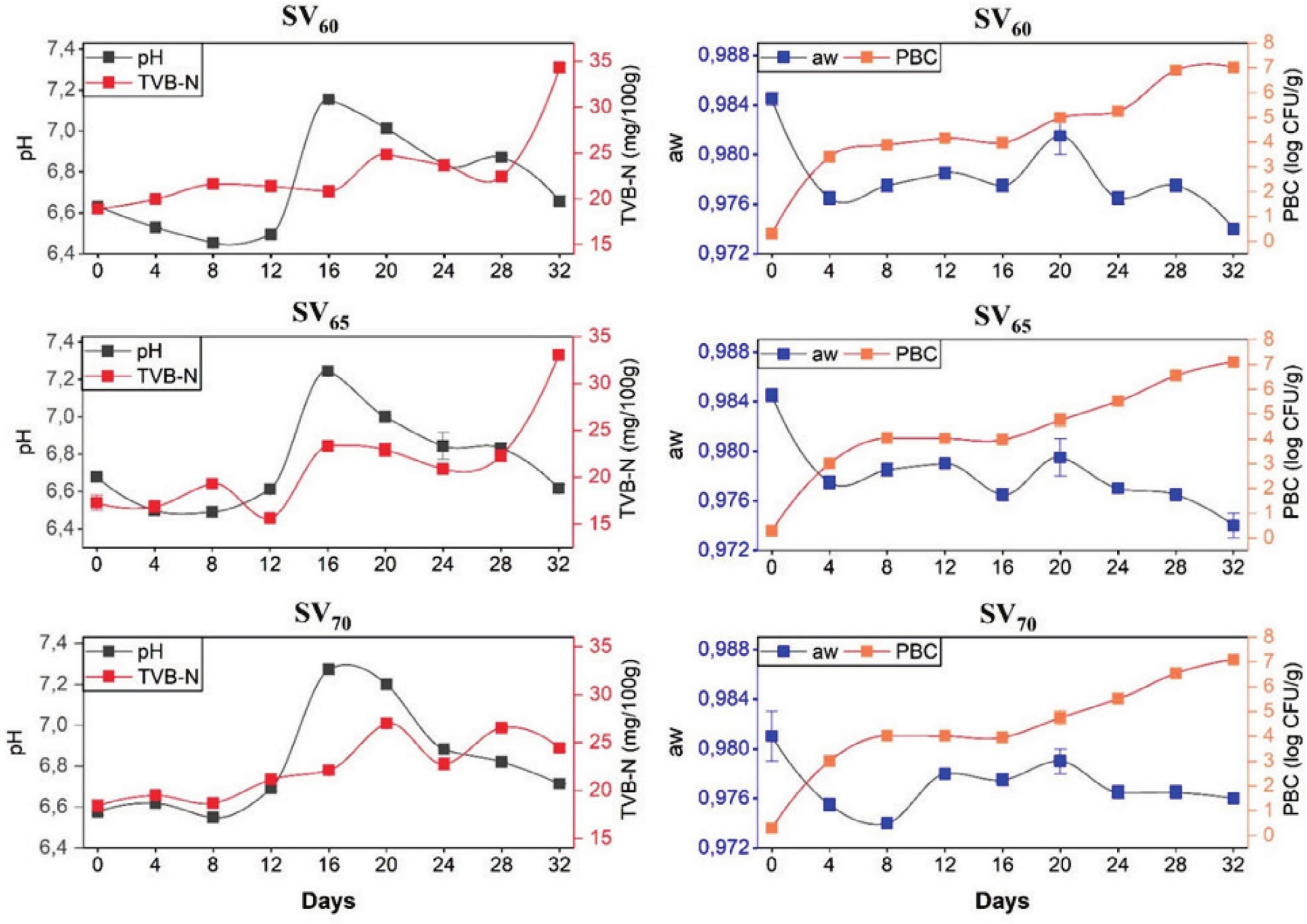
The relationship between pH and TVB‐N, and between water activity (aw) and psychrophilic bacterial count during storage in sous‐vide cooked fillets (SV_60_, SV_65_, SV_70_).

Water activity, an important factor in microbial activity, constitutes the portion of the total water content in food that is favorable for the growth and metabolism of bacteria, molds, and yeasts. Therefore, the microbiological stability of food can be measured by its water activity value. Microorganisms have a certain water activity value and cannot multiply below this value. For example, the growth limits for bacteria are 0.91–0.95 aw; for yeasts, 0.88 aw; for molds, 0.80 aw (Demirci 2012). The fact that all groups cooked sous‐vide showed a water activity value above 0.97 provided an ideal growth environment for mesophilic and psychrophilic bacteria during the increasing storage time, and the bacterial count increased steadily (Figure 5). Yeasts and molds, which are active at lower water activity levels compared to bacteria, remained at the same level until the 12th day of storage due to the high aw value in all groups (Figure 4). The increases in total yeast‐mold, total coliform, and anaerobic bacteria counts after the 12th day are thought to be due to the volatile basic substances formed by the breakdown of protein and non‐protein nitrogen compounds in fish meat, which increase the pH value and create the necessary environment for bacterial multiplication. Because, as can be seen in Figure 5, there is a linear relationship between the increase in pH value and the increase in TVB‐N value during storage. Chemical changes occurring in proteins during fish storage (breakdown of proteins into amino acids, breakdown of non‐protein nitrogen compounds) and the increase in volatile basic nitrogen substances also increase the pH value. The sharp increase observed after the 16th day in both pH value (Figure 1) and total coliform, total yeast, and mold, and total anaerobic bacteria counts (Figure 4) confirm this.

### 3.11 Sensory Evaluation

Groups SV_60_, SV_65_, and SV_70_ were evaluated by five experienced panelists based on taste and juiciness, odor, texture, aroma, and appearance parameters.

Fillets cooked sous‐vide at three different temperatures (60°C, 65°C, 70°C) were found to be similar in terms of taste and juiciness during the first 4 days (*p* > 0.05). During storage, taste and juiciness scores decreased in all groups, more markedly in group SV_60_ (Figure 5), and group SV_65_ performed better until day 20 (*p* < 0.05). After day 20, all three groups were similar in terms of taste and juiciness loss (*p* > 0.05). Studies have shown that the juiciness of sous‐vide cooked products decreases during storage (Díaz et al. 2009), and that the sous‐vide process applied at different temperatures (20 min at 75°C, 10 min at 85°C for 15 min, and 90°C for 10 min), the highest flavor scores were obtained in the groups cooked at 75°C, while the lowest scores were obtained in the groups cooked at 90°C (Çetinkaya et al. 2015).

Considering the microbiological analysis results, all groups were evaluated solely based on odor score after the 28th day. The panelists stated that they found the fillets to have a characteristic smoked fish odor. As shown in Figure 5, odor scores decreased during storage, remaining at a high level until the 24th day and then showing a significant decline thereafter (*p* < 0.05). As with the taste and juiciness evaluation, group SV_65_ received the highest preference in terms of aroma during storage. However, there was no statistically significant difference between the groups throughout the storage period (*p* > 0.05). Şişmanlar Altıkaya (2016) found the lowest odor scores on the 35th day of storage at the lowest temperature in a sous‐vide application performed at three different temperatures (60°C, 70°C and 80°C). González‐Fandos et al. (2005) also reported that the lowest odor scores were obtained on the 21st day in groups cooked sous‐vide at 65°C and on the 45th day in those cooked at 90°C. They reported that the resulting unpleasant odor could be related to bacterial and chemical changes during storage. Díaz et al. (2011) reported that odor and flavor scores in sous‐vide cooked salmon ( *Salmo salar* ) decreased by 60% after 10 weeks.

In terms of texture, groups SV_60_ and SV_65_ were found to be better than group SV_70_ on day 0 after sous‐vide processing (*p* > 0.05). Texture scores decreased in all groups during storage and were similar except on day 12, remaining at 7 or above until day 24 (Figure 5). At the end of storage on day 28, the texture scores were significantly reduced in all three groups, with no difference between groups (*p* > 0.05). Thus, the three temperatures applied in sous‐vide cooking (60°C, 65°C, and 70°C) had a similar effect on texture structure. Negative changes in the textural structure over time in sous‐vide cooked and cold‐stored fish have also been identified by other researchers (Díaz et al. 2009; Şişmanlar Altıkaya 2016; Erümit and Yıldız 2022).

**FIGURE 6:**
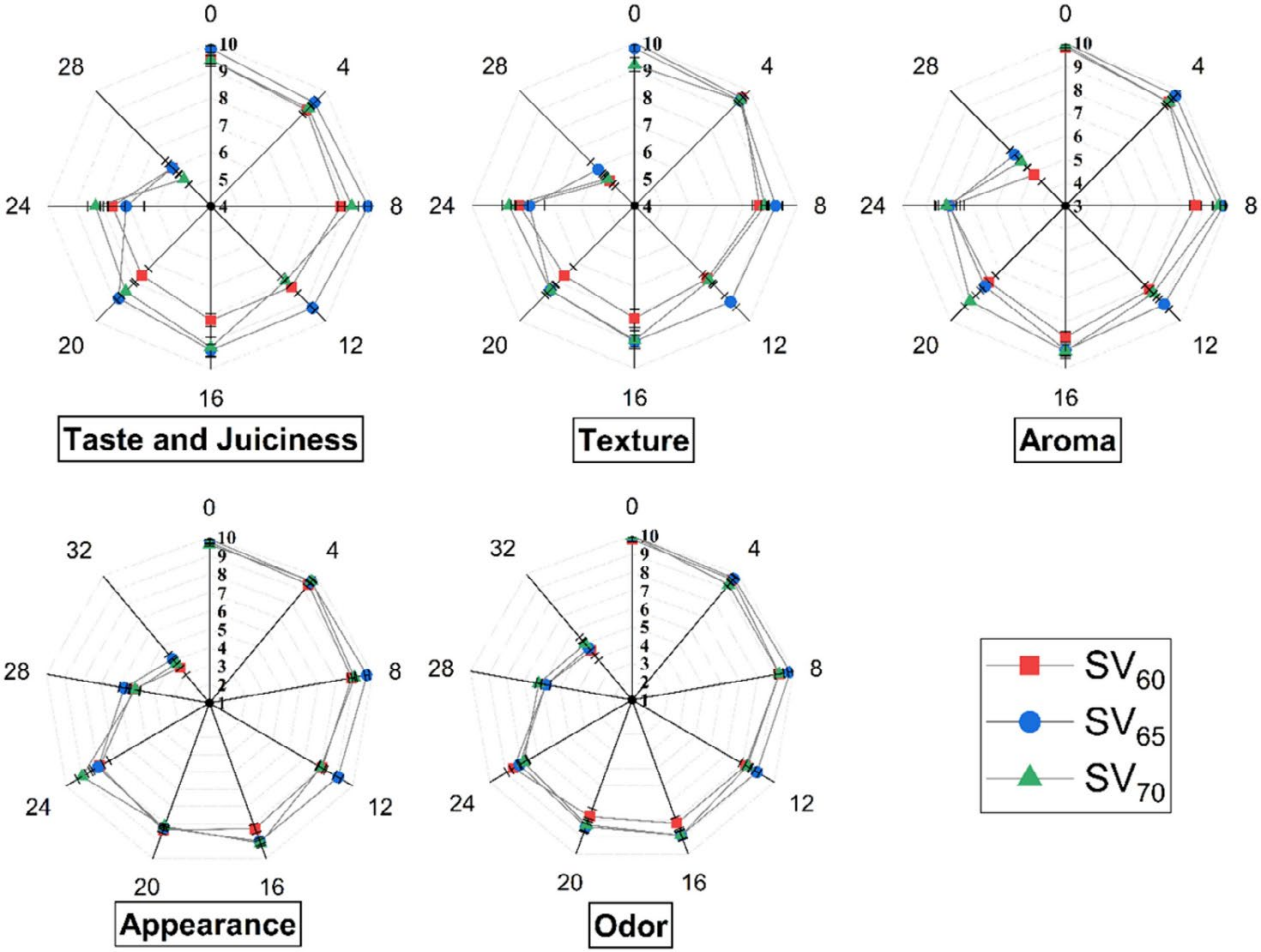
Sensory evaluation scores of groups SV_60_, SV_65_, SV_70_ during storage.

The liquid smoke aroma and the aromatic compounds developed during cooking were similarly perceived in sea bass fillets cooked sous‐vide at three different temperatures (*p* > 0.05). Aroma scores decreased in all groups except on day 8 due to volatile compounds developing alongside chemical and microbial changes associated with storage in the cooked product (*p* > 0.05). A sharp decrease was observed especially after day 24. Although the SV60 group had the lowest value at the end of the storage period, there was no statistically significant difference between the groups (*p* > 0.05) (Figure 6).

The appearance of the cooked product is a factor that directly affects the degree of product acceptance. Fillets cooked sous‐vide at three different temperatures were not found to differ in appearance. Physical (color and texture changes), chemical (color changes due to fat oxidation), and microbial changes (color and texture deterioration, stickiness, waterness, etc. due to microbial growth) occurring in the product during storage directly affect its appearance. The trend of decrease in appearance scores throughout storage was similar in all groups except on days 8 and 12 (*p* > 0.05). The appearance score decreased significantly in all groups after the 24th day of storage (Figure 5), but the groups did not differ from each other (*p* > 0.05).

Sensory parameters are directly influenced by the fish species used in studies, sous‐vide cooking temperatures, durations, and the use of additives. However, regardless of these parameters, the product is most appealing from a sensory perspective immediately after sous‐vide cooking. In trout cooked sous‐vide at different temperatures and durations (20 min at 75°C, 10 min at 85°C for 15 min, and 90°C for 10 min), the sous‐vide cooking process at 85°C achieved the highest appreciation in terms of color, appearance, aroma, and overall appeal (Çetinkaya et al. 2015). It has been stated that dried basil (0.5%), fresh garlic (0.25%), and dill (0.5%) in sous‐vide cooked fish may delay chemical and sensory deterioration (Ceylan and Ünal Şengör 2019), and rosemary and thyme extracts (5%) can improve the sensory quality of the product in terms of taste and smell (Bozova and İzci 2021). Lower pH, mesophilic and psychrophilic aerobic counts, and better sensory quality were obtained in mackerel (*Sarda sarda*) cooked sous‐vide at 70°C for 10 min with the addition of lemon juice (Coşansu et al. 2011). In rainbow trout treated with ground sage, sous‐vide processing yielded the lowest sensory values for color, odor, texture, and overall acceptability on day 45, and for appearance and juiciness on day 40. It was reported that sage treatment extended the shelf life and acceptability of the samples by at least 5 days (Çetinkaya 2020).

Sous‐vide products gradually lose their appeal criteria over extended storage periods due to chemical and microbiological changes. Díaz et al. (2009) stated that during the storage of salmon cooked sous‐vide at 80°C for 43 min, there was a loss of odor, taste, color and juiciness, as well as the emergence of undesirable odors and tastes. In another study involving salmon cooked sous‐vide at 80°C for 45 min, the same researchers reported significant sensory deterioration during refrigerated storage, including serious loss of cooked salmon odor and aroma, slight staleness, color deterioration associated with white sedimentation, and moderate loss of softness, chewiness, and juiciness (Díaz et al. 2011). They stated that sensory deterioration occurs before physical, chemical, and microbiological deterioration, and that microbiological quality alone could mislead the shelf life of sous‐vide cooked salmon (Díaz et al. 2011). Cropotova et al. (2019) investigated the effects of different sous‐vide cooking regimes (70°C and 80°C, 10 and 20 min), cold storage (0°C) and antioxidant use on the protein properties of Atlantic mackerel. They reported that cold storage duration caused a significant increase in protein oxidation, which led to fish meat hardening due to possible protein aggregation and conformational changes. However, they reported that the quality loss in mackerel samples cooked at 70°C was less than that in samples cooked at 80°C during cold storage. In this study, the high scores given for sensory parameters after sous‐vide cooking gradually began to decrease after approximately 2 weeks of storage. This was attributed to the increased microbial load and volatile nitrogen compounds released by the breakdown of large‐molecule compounds from the second week of storage onwards.

During sous‐vide cooking, the temperature and duration applied to the fish are crucial for the development of sensory characteristics and ensuring food safety. In this study, it was determined that the optimal temperature for preserving the sensory quality (taste and juiciness, aroma, texture and appearance) of sea bass flavored with liquid smoke during sous‐vide cooking is 65°C. This temperature is also confirmed by microbial analysis and TVB‐N results as the temperature at which microbial and chemical deterioration in sous‐vide sea bass can be delayed.

## 4 Conclusion

In this study, sea bass fillets that were flavored (salt + sunflower oil + liquid smoke) and sous‐vide‐cooked at three different temperatures (60°C, 65°C, and 70°C) were stored at 2°C and their physical, chemical, microbiological, and sensory properties were monitored during the cold storage. The combined flavoring imparted a pleasant aroma to the fish from an organoleptic perspective, while sous‐vide cooking preserved the nutritional content and quality to a significant extent. In conclusion, microbiological analyses were indicative in determining the shelf life of sous‐vide cooked liquid smoke flavored sea bass fillets. Considering all analysis results, it was determined that the optimal cooking temperature for sea bass cooked sous‐vide for 20 min with flavor (salt + sunflower oil + liquid smoke) is 65°C, and its shelf life is 24 days when stored at 2°C ± 1°C.

## Author Contributions

**Rukiye Köklü:** methodology, investigation, formal analysis, data curation, writing – review and editing. **Hülya Turan:** project administration, funding acquisition, supervision, validation, writing – original draft, writing – review and editing, conceptualization. **Can Okan Altan:** formal analysis, investigation, data curation. **Demet Kocatepe:** formal analysis, investigation, data curation, writing – review and editing. **Bayram Köstekli:** formal analysis, investigation, visualization, writing – review and editing. **Bengunur Corapci:** formal analysis, investigation, writing – review and editing.

## Funding

This research was financially supported by the Central Union of Aquaculture Producers (SUYMERBİR).

## Conflicts of Interest

The authors declare no conflicts of interest.

## Data Availability

The data that support the findings of this study are available from the corresponding author upon reasonable request.
